# Robust Long-Term Vehicle Trajectory Prediction Using Link Projection and a Situation-Aware Transformer

**DOI:** 10.3390/s24082398

**Published:** 2024-04-09

**Authors:** Minsung Kim, Byung Il Kwak, Jong-Uk Hou, Taewoon Kim

**Affiliations:** 1 School of Computer Science and Engineering, Pusan National University, Busan 46241, Republic of Korea; msungkim@pusan.ac.kr; 2 Division of Software, Hallym University, Chuncheon 24252, Republic of Korea; kwacka12@hallym.ac.kr (B.I.K.); juhou@hallym.ac.kr (J.-U.H.)

**Keywords:** intelligent transport system, situation-aware transformer, predictive model, trajectory prediction, deep learning

## Abstract

The trajectory prediction of a vehicle emerges as a pivotal component in Intelligent Transportation Systems. On urban roads where external factors such as intersections and traffic control devices significantly affect driving patterns along with the driver’s intrinsic habits, the prediction task becomes much more challenging. Furthermore, long-term forecasting of trajectories accumulates prediction errors, leading to substantially inaccurate predictions that may deviate from the actual road. As a solution to these challenges, we propose a long-term vehicle trajectory prediction method that is robust to error accumulation and prevents off-road predictions. In this study, the Transformer model is utilized to analyze and forecast vehicle trajectories. In addition, we propose an extra encoding network to precisely capture the effect of the external factors on the driving pattern by producing an abstract representation of the situation nearby the driver. To avoid off-road predictions, we propose a post-processing method, called link projection, which projects predictions onto the road geometry. Moreover, to overcome the limitations of Euclidean distance-based evaluation metrics in evaluating the accuracy of the entire trajectory, we propose a new metric called area-between-curves (ABC). It measures the similarity between two trajectories, and thus the accordance between the two can be effectively evaluated. Extensive evaluations are conducted using real-world datasets against widely-used methods to demonstrate the effectiveness of the proposed approach. The results show that the proposed approach outperforms the conventional deep learning models by up to 65.74% (RMSE), 60.13% (MAE) and 91.45% (ABC).

## 1. Introduction

**Figure 1:**
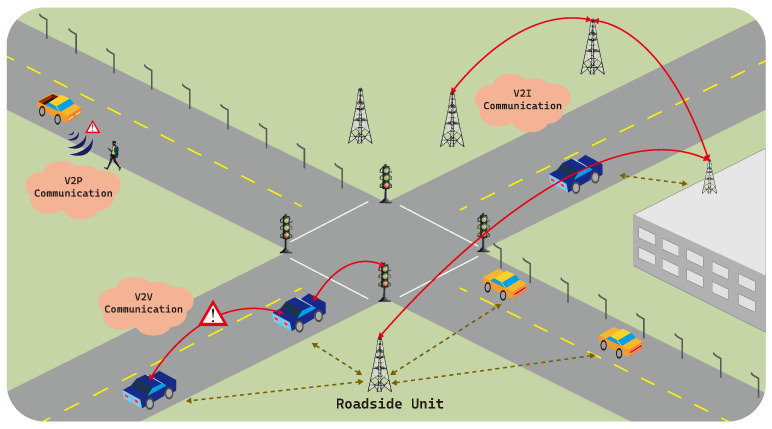
An illustration of smart city with intelligent transportation systems highlighting the communication-related core components, i.e., V2X communication and roadside units.

In the era of Intelligent Transportation Systems (ITS) [1,2,3] illustrated in Figure 1, accurate prediction of vehicle trajectories will play an important role in many cutting-edge applications [4], such as self-driving, platooning, collision prediction and smart traffic services. Inaccurate location estimations of vehicles can significantly degrade the quality of the applications and pose risk to both drivers and pedestrians. It is worth noting that the primary factors contributing to the risk of accidents include pedestrians, vehicles, road infrastructure and other factors on/nearby the road. Driving patterns are generally abstract descriptions of a driver’s vehicle manipulation [5] caused by the driver’s intrinsic driving habits, and it can be significantly influenced by diverse range of factors including even the ones that the driver cannot control. Therefore, learning drivers’ patterns to make accurate predictions can become much challenging especially in the complex urban road networks.

**Figure 2:**
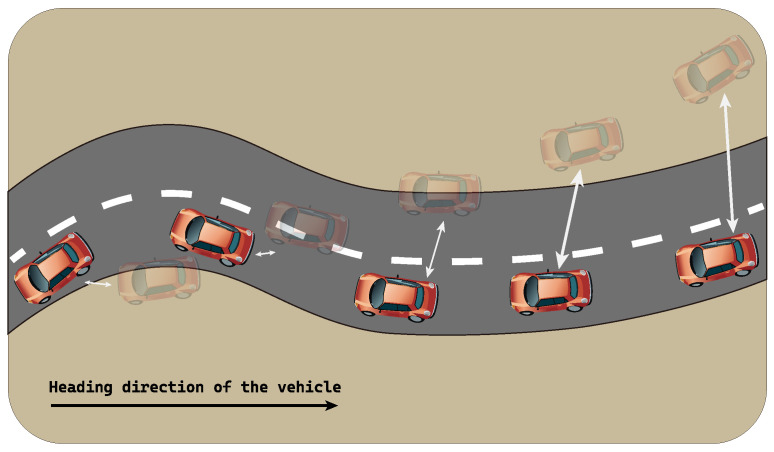
An example of the ground truth and predicted trajectory shown in solid and transparent cars, respectively, illustrating two major challenges in trajectory prediction research: (i) a small prediction error may result in off-road predictions, and (ii) the error may accumulate as the forecasting horizon increases.

Figure 2 illustrates two common difficulties in vehicle trajectory prediction studies. Let us assume that the car moves from left to right, and the solid and transparent cars correspond to the ground truth and predicted locations of the car, respectively. While the first, leftmost prediction deviates from the ground truth by only a small distance, it falls outside of the road boundaries, rendering it unsuitable for safety-critical applications such as self-driving or platooning. Furthermore, with an extended forecasting horizon (i.e., multi-step prediction), the accumulated error becomes more pronounced, leading to the following predictions significantly deviating from the ground truth.

Vehicle trajectory prediction is a learning task that involves generating possible future locations for a target vehicle by analyzing a time series dataset [6] containing past locations and/or other related information, such as velocity, acceleration and wheel control. In the general time series analysis tasks, conventional statistical approaches such as ETS and ARIMA have been widely employed to date [7]. However, recent advances and successes in deep learning have revealed that deep learning models can outperform the conventional approaches in complex domains [8,9,10]. In particular, recurrent neural network (RNN) [11] and their variants such as long short-term memory (LSTM) [12] and gated recurrent unit (GRU) [13] have been widely adopted in time series forecasting problems. Additionally, recent studies have shown that the Transformer model which was initially proposed to handle a large language model can forecast time series data with high accuracy [14].

Several state-of-the-art approaches have been proposed to forecast the vehicle trajectories under different scenarios [15,16,17,18,19], and they have achieved high accuracy in recognizing driving patterns and forecasting future trajectories. Despite such advancements, a significant challenge to overcome for it to be considered practical is *off-road prediction* [10]. It is common to leverage the geographic coordinate system (GPS)-based coordinates for training and inferring the vehicle locations. However, due to the scale of values involved, even a minor error in prediction can lead to substantial errors. For instance, the euclidean distance between two points, (40.500, 170.0) and (40.501, 170.0), corresponding to (40°30′00.0″ N, 170°00′00.0″ E) and (40°30′03.6″ N, 170°00′00.0″ E), respectively, is only 0.001. However, the physical distance between the two points in the real world amounts to 0.11 km, which is sufficient for a predicted point to deviate significantly from the actual road.

In addition, forecasting trajectories based on the learned driving pattern of a specific user requires extra attention. Common approaches involve learning the past trajectory to predict the future locations, which can be effective when the target area and road are already exposed to the model during training. However, if this is not the case, the trained model relies solely on the driving patterns latent in the past trajectories. It is important to note that driving pattern can also be influenced by external factors other than the driver’s intrinsic driving habits such as traffic control devices (e.g., traffic lights and speeding cameras), intersections and crosswalks to name a few. Thus, as pointed out in [19], high-precision trajectory forecasting becomes increasingly challenging. Although applying sophisticated models, such as hidden Markov models, artificial neural networks, Bayesian inference models, and machine learning, can enhance the prediction accuracy to some extent, capturing the nuanced patterns of minor vehicle movements with high accurately proved difficult. In a nutshell, such approaches facilitate the analysis of macroscopic trends within traffic flows, while achieving high-precision trajectory predictions from a microscopic perspective remains elusive. A recent study reported in [19] applied generative adversarial network along with a vehicle turning model to make predictions on complex urban roads. However, it does not take account for the external factors. Furthermore, limiting the performance evaluation metrics only to the Euclidean distance-based ones may overlook the possibility that the predicted trajectory falls outside the real road.

In this paper, we study and propose a multi-step vehicle trajectory forecasting scheme designed to be robust against error accumulation, preventing off-road prediction, and considering both the target driver’s driving pattern and the dynamic road situations such as traffic lights and speeding cameras. Compared to a single-step prediction, multi-step trajectory forecasting is challenging because the error accumulates as the forecasting horizon becomes longer. In addition, such errors can easily result in off-road predictions which makes the prediction less impractical. To address these two critical challenges, we propose a combined approach using the Situation-Aware Transformer (SAT) model followed by a link projection algorithm as a post-processing method. The Transformer model has demonstrated success in time series predictions, and in this study, we demonstrate its applicability in predicting multi-step vehicle trajectories with the aid of additional environment-encoding network. Additionally, the proposed link projection projects the predictions onto the actual road to further reduce the prediction errors.

Although the Transformer-based approach excels at capturing a particular user’s driving pattern, its application to the areas and roads encountered for the first time may not yield a realistic trajectory. One contributing factor is the presence of the diverse road structures and traffic control devices. To recognize the surrounding situations of an assumed driver and to produce realistic trajectory in response to such external factors, we propose to enhance the Transformer by adding an encoder network that abstracts the situations in the vicinity of the assumed driver. Finally, we propose a new performance evaluation metric tailored for long-term vehicle trajectory prediction. In general, the distance-based metrics such as root-mean-square error (RMSE) and mean absolute error (MAE) have been widely used to evaluate the performance in the trajectory prediction tasks. However, given the presence of the roads that vehicles must follow, two predicted locations yielding the same error distance may need to be taken differently depending on whether they are placed on the road or not. Also, the error measured in sample-by-sample manner, where a sample corresponds to a location of vehicle, does not fully capture the trajectory-wise accuracy, i.e., whether the predicted trajectory overlaps with the ground truth trajectory. In this regard, we propose a new metric that evaluates how much the line formed by the predicted trajectory deviates from the line drawn by the ground truth.

The summary of the contributions we make in this paper is as follows.

- We propose a long-term vehicle trajectory prediction method for complex urban roads. The proposed approach is robust to error accumulation and capable of capturing the driver’s driving pattern. To validate its effectiveness, we conduct comprehensive evaluations and performance comparison using the real-world vehicle trajectories.
- We propose an enhanced Transformer model to precisely forecast long-term trajectory of a vehicle. In particular, to capture the changes in the driver’s driving pattern in response to the external factors (e.g., traffic control devices), we propose to add an encoder network to the Transformer model which abstracts the situation nearby the driver.
- To assure the predicted trajectory lies on (or does not deviate from) the actual road, we propose a link projection scheme to project the prediction onto the link geometry.
- We propose a new performance evaluation metric tailored for vehicle trajectory prediction applications, called area-between-curves. It considers the similarity of the predicted trajectory to the ground-truth trajectory, gauging the agreement between the two patterns.

The remainder of this paper is organized as follows. In Section 2 we discuss the advantages and limitations of the related articles. In Section 3, we present the assumed system model and the proposed Situation-Aware Transformer model for the long-term vehicle trajectory prediction. In Section 4, we introduce the details about the dataset used in this study and the evaluation results. Finally in Section 5 we conclude this paper.

## 2. Related Work

The research on the vehicle trajectory forecasting has been carried out from diverse angles by using different approaches. In this section, we review the related studies that leverage deep learning models. Among the distinctive characteristics of the vehicle trajectory dataset, the most prominent feature is the temporal correlations among the consecutive data. Thus, the recurrent neural network (RNN) model and its variants, such as long short-term memory (LSTM) and gated recurrent unit (GRU), have been widely utilized in research for their strength in handling time series data.

Altché et al. [20] proposed an LSTM-based model for predicting vehicle trajectories. Their research utilizes a dataset collected from high-speed highways, and proposed method predicts vehicle trajectories for the next ten seconds. The average RMS error of the prediction is reported to be approximately 70 cm, surpassing the state-of-the-art techniques. However, the dataset used therein comprises highway segments, which are much different from the urban roads with complex traffic systems such as signal configurations. While LSTM neural networks exhibit strength in learning time-series data, there is a need for improvement to mitigate the accumulation of prediction errors, which may lead to trajectories deviating from actual roads or generating inaccurate predictions. Ip et al. [21] also conducted research using LSTM-based neural networks for predicting vehicle trajectories, but their approach differs from the general trajectory prediction. Instead of focusing on predicting the exact trajectory coordinates, they propose a method where the map centered around the vehicle is divided into specific-sized cells. Their research goal is to predict the next cell that the vehicle will visit. This approach aims to prevent the accumulation of errors in the overall trajectory by predicting the cells with potential drivability. Despite the advantages in avoiding cumulative errors, the possible limitation of this work is it cannot accurately predict the exact position of the vehicle.

While LSTM neural networks excel in handling long-term dependency in data, the capacity to retain information diminishes as the sequence length increases, resulting in the possible loss of data patterns. According to a research by Gers et al. [22], there is an indication of a linear increase in the cell states of LSTM networks during the processing of long-term sequence data, leading to inherent performance degradation issues. Long sequence time-series forecasting (LSTF) problems involve enhancing prediction capacity in response to longer sequences. Recently, Transformer models have shown superior performance in capturing long-range dependencies compared to RNN-based approaches, presenting significant potential for addressing LSTF problems. Accordingly, our study constructs a Transformer-based artificial neural network to address the issue of vehicle trajectory prediction. We introduce research related to vehicle trajectory prediction based on Transformer models, emphasizing their potentials in handling long-range dependencies and LSTF problems [23].

Quintanar et al. [24] conducted research on vehicle trajectory prediction by utilizing Transformer networks in various urban traffic scenarios, including intersections, highways, rotaries, etc. Experimental results showed that linear trajectories are correctly predicted but their proposed approach exhibited inaccuracies in circular intersection segments. This discrepancy appears to stem from both error accumulation and unawareness regarding the road, leading to incorrect predictions. While learning driving patterns for both rotational and straight segments is the primary goal in their research, the lack of solution to error accumulation in specific patterns is considered a limitation. Identifying a remedy for the accumulation of errors related to certain patterns remains unresolved. Some specific patterns of vehicle trajectories, such as turning segments and deceleration phases, have been identified as instances requiring overfitting, leading to generalization errors. However, if erroneous predictions persist even after learning linear segments preceding specific patterns, it may result in the accumulation of errors in the prediction, leading to further inaccuracies. Given the diverse driving patterns inherent in vehicle trajectories, addressing overfitting issues and error accumulation is crucial for a model to be robust. The accumulation of data based on various driving styles in vehicle patterns necessitates robust solutions to mitigate overfitting and error accumulation challenges.

Zhang et al. [25] proposed the Gatformer model based on the Transformer for predicting vehicle trajectories by considering the spatial-temporal interactions among traffic agents. In their model, images of traffic scenes are learned and encoded to perform predictions using the Transformer encoder-decoder. Experimental results using the Lyft dataset demonstrate superior performance in terms of prediction accuracy and inference time compared to the models under comparison. While the Gatformer is shown to be useful for multi-vehicle trajectory prediction, a limitation of this study is its applicability to long-range trajectory prediction. The model may not be suitable for predicting long-distance trajectories as it primarily learns vehicle patterns from dash cam footage, which may not capture the necessary information for long-range predictions such as the precise coordinate of the vehicle. Despite the overarching challenge of trajectory prediction, the diverse traffic environments and variations in road traffic systems across countries may pose difficulties in learning robust patterns.

In this study, we take the Transformer model as a basis for making multi-step trajectory predictions. To minimize the error accumulation and to avoid off-road predictions, we propose a link projection algorithm to project the predicted coordinate on to the nearest link geometry. In addition, to capture the extra factors affecting the user’s driving pattern, we propose to put an encoder network on top of the Transformer that abstracts the traffic situation surrounding the driver.

## 3. Proposed Idea

In this section, we introduce the proposed high-precision vehicle trajectory prediction method in detail. In particular, Section 3.1 introduces the proposed SAT model, and Section 3.2 explains the proposed link projection algorithm which effectively reduces the error accumulation in the long-term trajectory prediction.

### 3.1. Situation-Aware Transformer

**Figure 3:**
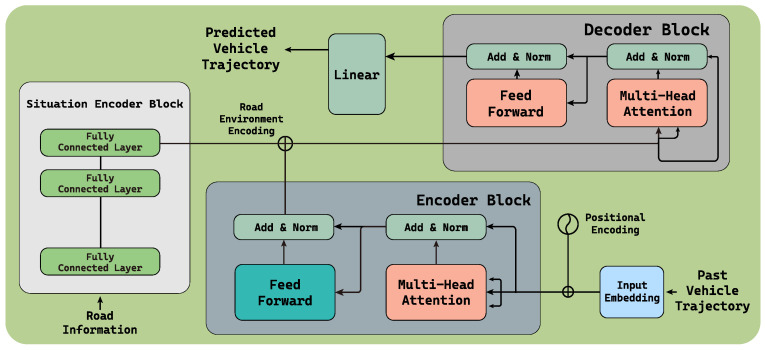
The proposed situation-aware Transformer model which leverages the encoded road information along with the recent trajectory to predict long-term trajectory with a high degree of accuracy.

The goal of the proposed trajectory prediction model is to make realistic and precise vehicle trajectory prediction with a specific emphasis on minimizing multi-step prediction errors and capturing the user’s driving pattern changes caused by the various elements of the traffic system installed on the roads. The proposed model, called SAT, is an enhanced Transformer model, particularly designed to achieve the goal. The proposed design of the model is visualized in Figure 3, consisting of the following blocks.

- **Encoder block** reads the past vehicle trajectory of which length, called look-back window size, is $N_{lb}$. This block encodes the recent trajectory of the target vehicle to extract the driving pattern. The encoded pattern is passed to the following decoder block to generate the future trajectory.
- **Situation encoder block** is the newly attached block to the Transformer model, whose goal is to understand the situation on the road in the vicinity of the target driver. In this study, the existence of the intersections and traffic control devices are considered as the situation. In addition, other factors that can affect the driving pattern are also considered. For example, in this study, the dataset we collected is the bus trajectory, and thus the location of the bus stops is used as an input to the situation encoder block as well. This entails a preselection of elements that influence the driver’s driving patterns, and the information is conveyed to the situation encoding block in vector form. Each element is assigned to a specified position within the vector, reflecting the presence of elements. Considering the heading direction of the target vehicle, the existence of a traffic light, traffic enforcement camera, intersection, and bus stop within the range of *R* meter is binary encoded, and then passed to the situation encoder block. If there are multiple instances of the same element, the nearest one will be processed first. In this study, a multi-layer perceptron (MLP) is used to construct the situation encoder block.
- **Decoder block** receives the information regarding (i) the abstract representation of the situation from the situation encoder block, and (ii) the encoded trajectory of the vehicle from the encoder block, and then generates the future trajectory (i.e., location/position of the target vehicle). The attention layer at the bottom of the decoder block concatenates the encoded vehicle pattern and the recognized situation.
  In contrast to the RNN and its variants (e.g., LSTM and GRU), the Transformer model inherently lacks the concept of sequence or order, making it challenging to preserve the temporal relationships of the data when processing time-series data in parallel. Therefore, position encoding is used to preserve the spatiotemporal correlations among the input data in our study. Additionally, the combined information considers both the driver’s recent driving pattern and the surrounding road situation, and it is utilized to predict a high-precision future trajectory.

The input to the proposed SAT model is the concatenation of the past history of length $N_{lb}$ and the binary-encoded vector that indicates whether or not a traffic light, intersection, traffic enforcement camera and bus stop is located nearby in the heading direction. As discussed in Section 1, due to the scale of the GPS coordinates, a tiny change in GPS coordinate amounts to a large distance in meter units. Thus, as part of the pre-processing, we applied a scaling operation on longitude and latitude separately so that the scales of the GPS coordinates become relatively larger, making it easier to correctly predict the future coordinates. The information regarding the road situation can be automatically retrieved if the supporting infrastructure is provided. For example, Seoul, the city and capital of the Republic of Korea, provides such information via the T-Data web service [26]. One can easily access the accumulated trajectory logs, real-time driving vehicle information and additional information regarding the situation by using a simple HTTP interaction with the web service.

### 3.2. Link Projection

As long as the prediction is imperfect, which is almost always the case in practice, prediction error occurs. One may be able to minimize the error for a single-step prediction, but as the forecasting horizon becomes larger, the error accumulation is inevitable. Such error accumulation not only degrades the accuracy of the predicted trajectory, but also causes off-road prediction, which can be fatal in safety-critical applications. One may minimize the single-step and multi-step errors by over-fitting the model, but in this paper, we propose a general solution called link projection that effectively prevents off-road predictions at low cost.

The link projection method is inspired by the geometric concept called perpendicular foot which denotes the intersection point formed when a line segment, denoted as *L*, is intersected perpendicularly from a vertex, *P*. In this study, we adopts an advanced variation of it by proposing a novel approach of creating an intersection point. Instead of generating an intersection point directly, the line segment *L* is further segmented into 1-m-interval sub-segments to produce vertices within the road geometry. Subsequently, a perpendicular foot is projected from each vertex to correct errors resulting from deviations outside the road boundary. This process entails identifying the vertex closest to the projected perpendicular foot and adjusting its position by the discrepancy measured from the original location. This method significantly reduces prediction errors and improves the alignment accuracy of vertices with the actual road geometry.

**Figure 4:**
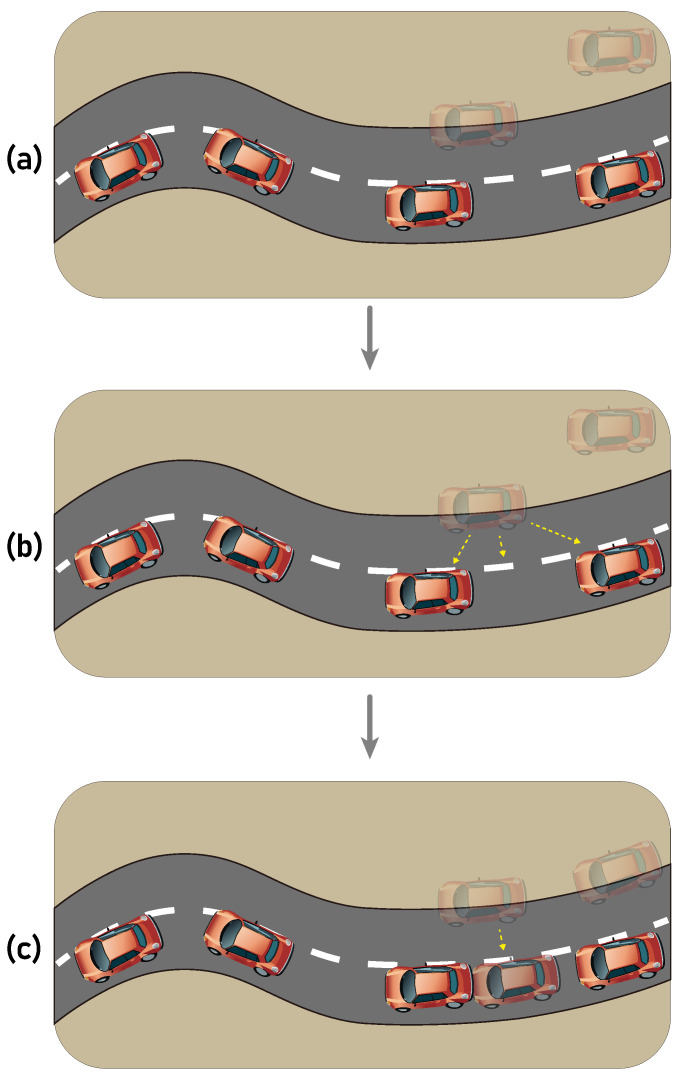
An example scenario illustrating the link projection method we propose to prevent error accumulation and to prevent off-road prediction. In the subfigures (**a**–**c**), transparent and solid objects represent the predicted and ground truth locations of a vehicle, respectively. The subfigures (**a**–**c**) in sequence demonstrate the operation and advantage of the proposed link projection.

An illustrative description of the proposed algorithm is shown in Figure 4. The car in the figure is assumed to be moving to the right, and the assumed task is to predict the third and fourth position of the car. As it can be seen in Figure 4a, the first prediction (denoted by a transparent car image) is slightly deviated from the actual location (denoted by a solid car image. However, due to the error in the first prediction, the second prediction is further away from the ground truth, causing error accumulation. Figure 4b,c sequentially show the proposed link projection. When the prediction has been made by the proposed SAT model, the link projection is invoked and it tries to project the predicted location onto the link geometry (i.e., the actual road) with the shortest distance. Such a post-processing method shifts the predicted location to the on-road position, and thus the prediction error can be minimized while correctly placing the prediction on the actual road. Also, the reduced error of the first prediction reduces the error in the following predictions as can be seen at Figure 4c.

The Algorithm 1 describes the overall procedure of the proposed approach with link projection. There are three input arguments to the algorithm: *T*, the past trajectory with a length of $N_{lb}$ (i.e., the look-back window size), *N* is the forecasting horizon length, and *L* is the set of the road segments. The proposed algorithm maintains a queue internally to store both the past and predicted trajectory, which is initialized with *T* (line:1). For each iteration (lines:2–6), SAT is called to make a single-step prediction (line:3). The predicted location is then projected onto the nearest road, and pushed to the tail of the queue (lines:4–5). The proposed algorithm returns the predicted trajectory of which size is *N*. The first prediction is completely based on the past trajectory. On the other hand, the second and the following predictions are partially based on the predicted values. Due to the proposed link projection method, the prediction error is diminished on each iteration and thus, the accumulated errors in the following predictions are also reduced.
**Algorithm 1:** Link Projection-Based N-Step Trajectory Predictions
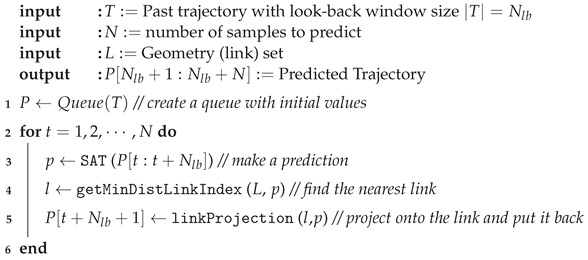


### 3.3. Overall Procedure

**Figure 5:**
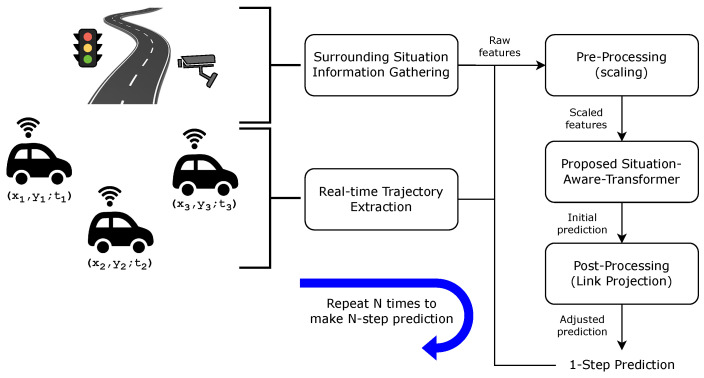
Illustration of the overall procedure proposed in this paper where $\left(x_{i},y_{i};t_{i}\right)$ corresponds to *x* and *y* coordinate collected at time $t_{i}$. The procedure repeats N times to make N-step prediction.

Figure 5 illustrates the overall procedure of the proposed approach. After gathering the information which is the concatenation of the $N_{lb}$ number of past trajectory data points and the extracted situation information, the min-max scaling operation is carried out on the input data during pre-processing. The processed information is then passed to the SAT to make an accurate prediction on the future trajectory data points. To further enhance the prediction accuracy while guaranteeing the on-road prediction, link projection is carried out as a post-processing method. The procedure is repeated *N* times to generate *N* data points as a future trajectory.

## 4. Evaluation

In this section, we discuss the details about the dataset in Section 4.1, the applied evaluation metrics in Section 4.2, and the evaluation and comparison results in Section 4.3.

### 4.1. Dataset

One of the widely used datasets for the vehicle trajectory prediction studies is NGSIM [27]. However, it primarily focuses on highway scenarios, which is different from the present study. Therefore, it can be used to learn the driving patterns on low-complex roads, given its highway-centric nature [28]. However, due to the high complexity of the urban scenarios caused by the complex structure of the road (e.g., rotational segments and intersections) and the traffic systems, NGSIM has limitations in learning complex driving patterns on urban roads. The Republic of Korea, with its relatively small territory considering its population, features a variety of urban road scenarios with diverse road shapes and patterns. This characteristic offers an advantage in evaluating the performance of the trajectory prediction models under complex scenarios. In this study, the real-time bus trajectory of a particular bus operating in the Seoul city [29] was collected and used for performance evaluation. The bus dataset includes the speed, direction (i.e., wheel angle), altitude, etc. with GPS coordinates based on the EPSG:4326 coordinate reference system [30].

In addition, as mentioned earlier, learning driving patterns solely from the vehicle trajectory is challenging because of the external factors affecting the driving patterns such as traffic control devices. Therefore, we have incorporated such features into our training set, which will also be passed to the proposed SAT. The utilized data includes the location information of traffic signals, traffic enforcement cameras, intersections, and bus stops. The reason for using such data is its significant impact on vehicle deceleration. When a driver slows down the vehicle by encountering such elements, the intervals between consecutive data points in the trajectory become narrower. On the other hand, when the driver accelerates the vehicle, the intervals are expected to widen [31]. On a linear segment of the road, the intervals will remain almost constant in general. The presence of such dynamic or slight changes in data intervals can have a significant influence on driving patterns, which is why the external factors are considered in this study to generate the realistic trajectories.

The utilized dataset consists of route trajectories recorded by an operating bus between 1:00 PM and 2:00 PM on 1 October 2023. The average time interval between the two consecutive data samples is approximately fifty seconds. The vehicle trajectory dataset includes 526 data points of vehicle coordinates, which were then split into an 80:20 portion for training and test, respectively.

### 4.2. Evaluation Method

In this work, we employ three evaluation metrics, RMSE, MAE, and the Area Between Curves (ABC), where the first two are the widely used ones for evaluating the performance of the trajectory prediction models and the last one is what we propose in this paper. Let z=(x,y) be the ground truth location of a vehicle, whereas let $z^{\prime }=\left(x^{\prime },y^{\prime }\right)$ be the predicted counterpart. The widely used performance metrics, RMSE and MAE, are based on the Euclidean distance between the two points *z* and $z^{\prime }$, defined as $\sqrt{{\left(x-x^{\prime }\right)}^{2}+{\left(y-y^{\prime }\right)}^{2}}$. To be specific, RMSE and MAE are defined as follows assuming there are *n* number of *z* and $z^{\prime }$ pairs:

- **RMSE** is one of the representative standard statistical metrics indicating the difference between predicted values and actual values, defined as follows:
  (1) $$\mathrm{RMSE}=\sqrt{\frac{1}{n}\sum_{i=1}^{n}{\sqrt{{\left(x_{i}-x_{i}^{\prime }\right)}^{2}+{\left(y_{i}-y_{i}^{\prime }\right)}^{2}}}^{2}}$$
- **MAE** is also one of the statistical metrics for evaluating the difference between predicted values and actual values. MAE is considered a robust metric, particularly in the context of coordinate data, as it is sensitive to small decimal places. MAE is less affected by outliers, making it a robust evaluation metric.
  (2) $$\mathrm{MAE}=\frac{1}{n}\sum_{i=1}^{n}\left|\left(x_{i}-x_{i}^{\prime }\right)+\left(y_{i}-y_{i}^{\prime }\right)\right|$$

**Figure 6:**
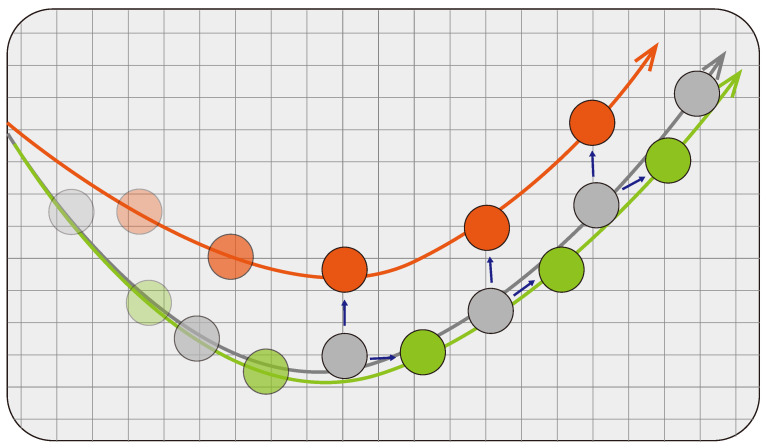
Illustration of the ground truth trajectory (gray) and two predictions (orange and green) with the same degree of error. The blue arrows indicate the error distance which is the same between the two predictions. Utilizing the Euclidean distance for model evaluation may not distinguish between the prediction with better pattern agreement (green) and the worse one (orange).

Figure 6 shows an illustrative example showing the ground truth trajectory (gray circles) and the two predicted trajectories (green and orange circles).

For a particular ground truth data point, its predictions are assumed to be away from it by the same magnitude. Thus, by using the Euclidean distance-based metrics, both predictions are considered comparable. However, if one considers ow much the predicted trajectory overlaps with the ground truth (i.e., pattern agreement), the two predictions, orange and green, can be treated differently. This simple example shows the limitation of the distance-based evaluation metrics in evaluating the trajectory-wise pattern accuracy. As a result, we propose a new performance evaluation metric in this paper, called ABC:

- **ABC** (Area Between Curves) algorithm is a path difference measurement technique proposed in this paper. The path similarity cannot be measured by distance-based metrics. However, in the case of trajectory prediction problem, it is important to produce the trajectory that overlaps with the ground truth as much as possible to achieve the driving pattern agreement. The proposed ABC draws a curve by the ground truth and another by the predictions. Then, it measures the area of the closed region formed by the two curves, which can be done by counting the number of pixels belonging to the closed area.

The proposed ABC is not intended to be used as the sole evaluation metric for trajectory prediction problem. However, when used with other metrics such as RMSE and MAE, ABC can reveal additional information regarding whether or not the predicted trajectory align with the ground truth. The prediction yielding a smaller closed area indicates better pattern agreement. The Algorithm 2 details the procedure of the proposed ABC algorithm. Given the set of predicted trajectory points *P* and the ground truth *G*, the ABC algorithm first draws separate lines (lines:1–2) along the respective data points. Then, it finds the closed regions formed by the two lines. (line:3). The closed region of the intersection area is filled with black color and then converted into an image (line:4). The image is then converted to a 2D array (line:5) so that each pixel in the image can be iteratively visited. For each pixel, if its color is black, meaning that it belongs to the closed region, the counter $c_{bp}$ is increased by one. The $c_{bp}$ is returned from the algorithm to notify the number of pixels included in the closed region formed by the two lines.
**Algorithm 2:** Area Between Curves (ABC)
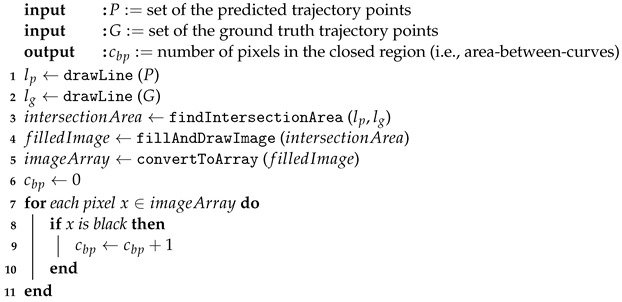


In this study, we evaluate the performance of the considered models from two perspectives. The RMSE and MAE metrics assess the errors in distances on the Euclidean space, while the ABC metric evaluates the morphological similarity between the two trajectory curves drawn by the ground truth and the predicted data. Both RMSE and MAE are used to evaluate the errors in s sample-by-sample manner, where each sample corresponds to a particular geographical location of the vehicle. Thus, such measures assess the accuracy of the predicted individual locations. However, they are not suitable for assessing whether or not the entire trajectory from predicted samples overlaps with the ground truth trajectory. To evaluate the accuracy of the entire trajectory (i.e., driving pattern agreement), not each individual sample, we have proposed ABC metric which counts the differences between the two entire trajectories.

### 4.3. Evaluation and Comparison Results

The evaluations were carried out by measuring the performance metrics discussed in the previous section. For the performance comparison, we have implemented and evaluated the following models which are widely used deep learning models for trajectory/time-series prediction. We used a common set of hyper-parameters/configurations, i.e., a look-back window size of 8, an Adam optimizer with a learning rate 0.001, and min-max scaling applied during pre-processing.

- **Vanilla LSTM**: It is well-known for effectively learning long-term patterns in sequential data, which can be done with a relatively simple architecture. The learned information is stored in the cell state, and the addition or deletion of information occurs through gates. This model has gained prominence for its ability to capture long-term dependencies in sequences and is widely utilized in various applications including natural language processing, speech recognition, and time series prediction. Each LSTM unit incorporates a cell state and three gates: input, forget, and output, effectively managing the flow of information while maintaining essential temporal relationships within the network.
- **1D-CNN**: It can efficiently capture temporal patterns, offering superior performance in predicting the future and analyzing traffic behaviors. This approach significantly enhances the accuracy and efficiency of vehicle trajectory prediction by enabling in-depth time-series analysis without the need for complex feature extraction processes. In our experiment, the 1D-CNN model was constructed with 64 convolutional filters. The resulting values were further processed by flattening the output for dense layers, culminating in a final prediction layer with two outputs to forecast (x,y) coordinates each.
- **ConvLSTM**: It integrates spatial and temporal features to learn vehicle trajectories, offering high accuracy in predicting future positions and recognizing behavior patterns. This architecture captures subtle spatio-temporal correlations even in complex road environments and traffic flows, significantly enhancing the reliability of vehicle trajectory analysis. In this study, the ConvLSTM model was structured with a ConvLSTM2D layer utilizing 64 filters, which is followed by a sequence of flattening and dense layers, culminating in the final layer that predicts two distinct features.
- **Vanilla Transformer (Vanilla TF)**: With its unique attention mechanism, the Transformer model can effectively capture the temporal correlation among consecutive features in vehicle trajectory, excelling in predicting future vehicle locations and recognizing complex traffic patterns. This approach can outperform traditional sequence learning methods by capturing deeper temporal dependencies, offering significant advantages in the analysis of vehicular movements. According to recent studies, the Transformer model was constructed using a series of normalization and attention mechanisms, initiated by layer normalization and multi-head attention for processing inputs. This configuration is enhanced by feed-forward networks comprising Conv1D layers for further transformation, following the principle of self-attention across multiple heads to effectively capture dependencies without the constraints of sequence alignment.
- **Situation-Aware Transformer (SAT)**: The Transformer-based model we propose in this study is further enhanced to effectively adapt to dynamic road situation to precisely predict vehicle trajectories considering both the learned driving patterns and the surrounding situation. This proposed model is based on the encoder-decoder model of the vanilla Transformer, and then enhanced by introducing an additional encoder for understanding surrounding road situations such as traffic control devices, intersections, and bus stops.
- **Situation-Aware Transformer with Link Projection (SATLP)**: The proposed SAT model followed by the link projection operation to correct the prediction error.

The Table 1 summarizes the RMSE performance of the models considered in this paper, where the one that outperforms most is marked bold on each row. The proposed SATLP achieved the best performance in most scenarios. However, the proposed SATLP ranks third in the case of the straight lane scenario, where the performance gap is negligible. The performance enhancement of the SATLP model, particularly in non-straight scenarios, stems from the incorporation of traffic information such as the presence of an intersection, traffic light, bus stop, etc., suggesting that driving patterns are affected by such surrounding situation. In contrast, the straight lane scenario typically involves more consistent driving patterns, which can also be effectively accomplished with a simpler pattern learning model. However, in other scenarios, the variability in driving patterns is heavily influenced by traffic information, and there is also a cumulative error component to consider, which justifies the enhanced performance of SATLP that employs Link Projection.

Table 2 summarizes the MAE performance of the considered models. Given that both RMSE and MAE are based on Euclidean distance for measuring errors, the results reported in the table are similar to those in Table 1. Consequently, it is observed that the outcomes of RMSE and MAE evaluations are closely aligned. However, among the results from RMSE and MAE, the latter provides more intuitive outcomes that can be easily interpreted on the real-world, meter-unit coordinate system. This is because RMSE involves squared and square-root terms, which can obscure the intuitive understanding of the results. In contrast, MAE calculates the error as the absolute value of the distance difference, making the error more straightforward to understand.

In this paper, we propose a new performance evaluation metric, ABC, to evaluate the similarity between two trajectories. Table 3 shows the ABC performance of the considered approaches, and the reported values are computed by comparing each predicted trajectory with the ground truth. The ABC evaluation is conducted on images with a resolution of 1920 × 1080, totaling 2,073,600 pixels. Please note that ABC first fills in the closed area formed by the line drawn by the ground truth vehicle coordinates and another line drawn by the predicted value with the black colors. Then, by counting the number of black pixels, the level of dissimilarity is gauged. In contrast to the previous RMSE and MAE performance, the proposed SATLP outperformed the rest across all scenarios with respect to ABC performance metric. This shows that the predicted trajectory produced by SATLP matches the ground truth trajectory most. Comparative analysis between the Vanilla TF model without the situation encoder and the SAT model with the situation encoder, indicates an approximate performance improvement of 71.76% in the Entire Trajectory scenario. This suggests that providing the situational road information to the Transformer model can effectively enhance the prediction performance by considering the effect of the dynamic situation on the driving pattern. Please note that the level of the magnification of the scene is different from one scenario to another, and thus the reported values on one row cannot be compared to the numbers on the other rows as they are.

**Figure 7:**
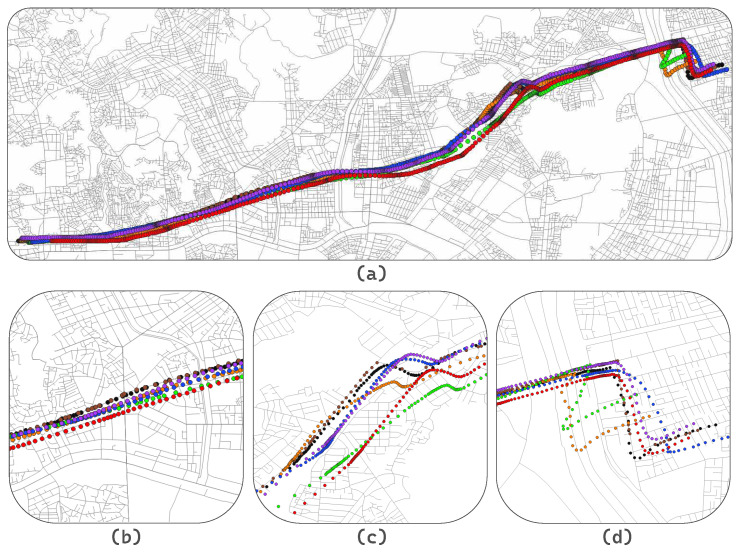
Visualization of the trajectories produced by the considered models on different scenarios: (**a**) entire trajectory, (**b**) straight lane, (**c**) curved lane, and (**d**) intersection, where blue dots correspond to the LSTM trajectory, orange dots are for 1D-CNN, green dots are for ConvLSTM, red dots are for Vanilla Transformer, purple dots are for SAT, brown dots are for SATLP, and black dots are the ground truth.

Figure 7 depicts the location of the actual vehicle (i.e., ground truth) and the predicted ones in a single figure. In addition to the aforementioned evaluation metrics, i.e., RMSE, MAE and ABC, the figure visually shows if the predicted trajectory is laid on the actual road. This visualization serves to highlight the limitations of the evaluation methods by demonstrating the effectiveness of the proposed approach in trajectory prediction and its practical applicability in real-world scenarios. Figure 7a illustrates the entire trajectory of the ground truth on the map, and the individual trajectories produced by the considered models. Although it is difficult to differentiate one trajectory from the rest, it can be seen from the figure that the Vanilla Transformer (red) and ConvLSTM (green) deviate significantly from the ground truth (black) in the middle portion of the trajectory. Also, at the end of the trajectory, both 1D-CNN (orange) and ConvLSTM (green) differ markedly from the ground truth. Those two segments on the trajectory have relatively complex road structure and have many traffic control devices along with intersection and bus stops. Due to such high complexity, some models have yielded low accuracy in prediction.

As observed in Figure 7b which highlights one of the linear segments on the trajectory, all the considered models do not significantly deviate from the road geometry and the ground truth in straight sections due to the simplicity of the road segment. On the other hand, Figure 7c demonstrates that errors accumulated from the previous straight line segment have resulted in a substantial deviation from the road geometry. Also, the complexity of the road structure caused more errors. Despite such challenges, the proposed SATLP minimizes the errors in trajectory, validating its effectiveness. The similar trend repeats in Figure 7d as well. While all the considered models have successfully learned the driving patterns in making turns, there still is a continuous accumulation of errors. As a result, most of the models deviate much from the ground truth which is not the case to SATLP. Despite the complex structure of the road or the dynamically changing driving route, the proposed SATLP has successfully demonstrated a consistent error-minimizing performance while overlapping its trajectory much with the ground truth and the road geometry.

## 5. Conclusions

In this study, we have proposed a situation-aware artificial neural network model to predict vehicle trajectories. The latter have previously been studied using statistical and machine learning approaches. To make accurate predictions especially on complex urban roads, we introduced a Transformer-based approach that also considers the situation around the driver by incorporating an additional encoder network. Furthermore, to avoid off-road predictions, we have proposed a link projection technique that projects the predictions onto the road geometry. To overcome the limitations of the conventional Euclidean distance-based metrics in assessing the accuracy of the entire trajectory, we have proposed an ABC metric that measures the similarity between two entire trajectories, thus effectively assessing the accuracy of the entire trajectory on a macroscopic scale. Through extensive evaluations conducted on real-world datasets, our model with link projection has significantly outperformed conventional approaches, achieving improvements of up to 65.74% (RMSE), 60.13% (MAE), and 91.45% (ABC) on the entire trajectory scenario.

## Figures and Tables

**Table 1:** Performance comparison with respect to RMSE measured in meters. Bold denotes the values with the best performance in the given category.

Scenario	LSTM	1D-CNN	ConvLSTM	Vanilla TF	SAT	SATLP
Intersection	0.0539	0.1060	0.0525	0.0344	0.0443	**0.0315**
Straight Lane	0.1433	0.1216	**0.0553**	0.2367	0.0710	0.0791
Curve Lane	0.0753	0.1773	0.2196	0.0606	0.0676	**0.0588**
Entire Trajectory	0.1302	0.1010	0.0878	0.2046	0.0748	**0.0701**

**Table 2:** Performance comparison with respect to MAE measured in meters. Bold denotes the values with the best performance in the given category.

Scenario	LSTM	1D-CNN	ConvLSTM	Vanilla TF	SAT	SATLP
Intersection	0.0368	0.0896	0.0389	0.0312	0.0374	**0.0271**
Straight Lane	0.1063	0.0853	**0.0401**	0.1721	0.0524	0.0589
Curve Lane	0.0646	0.1416	0.1954	0.0526	0.0542	**0.045**
Entire Trajectory	0.0988	0.0708	0.0597	0.1427	0.0617	**0.0569**

**Table 3:** Performance comparison with respect to ABC which counts the number of pixels that are belonging to the closed area formed by the ground truth and predicted trajectories. The reported values are the pixel count, indicating the difference between the two trajectories. Bold denotes the values with the best performance in the given category.

Scenario	LSTM	1D-CNN	ConvLSTM	Vanilla TF	SAT	SATLP
Intersection	562,145	960,666	979,646	918,796	617,366	**524,391**
Straight Lane	108,663	136,888	169,647	315,519	66,964	**10,734**
Curve Lane	1,308,515	1,462,938	1,228,900	702,028	486,265	**233,317**
Entire Trajectory	42,530	51,494	93,393	114,687	32,384	**9804**

## Data Availability

The data presented in this study are available on request from the corresponding author.
